# Novel Adjuvant S-540956 Targets Lymph Nodes and Reduces Genital Recurrences and Vaginal Shedding of HSV-2 DNA When Administered with HSV-2 Glycoprotein D as a Therapeutic Vaccine in Guinea Pigs

**DOI:** 10.3390/v15051148

**Published:** 2023-05-10

**Authors:** Sita Awasthi, Motoyasu Onishi, John M. Lubinski, Bernard T. Fowler, Alexis M. Naughton, Lauren M. Hook, Kevin P. Egan, Masaki Hagiwara, Seiki Shirai, Akiho Sakai, Takayuki Nakagawa, Kumiko Goto, Osamu Yoshida, Alisa J. Stephens, Grace Choi, Gary H. Cohen, Kazufumi Katayama, Harvey M. Friedman

**Affiliations:** 1 Infectious Disease Division, Department of Medicine, Perelman School of Medicine, University of Pennsylvania, Philadelphia, PA 19104-6073, USAjohn.lubinski@pennmedicine.upenn.edu (J.M.L.); tzel2395@gmail.com (B.T.F.); alexis.naughton19@gmail.com (A.M.N.); lhook@pennmedicine.upenn.edu (L.M.H.); kevinpe@pennmedicine.upenn.edu (K.P.E.); 2 Pharmaceutical Research Division, Shionogi & Co., Ltd., Osaka 561-0825, Japan; masaki.hagiwara@shionogi.co.jp (M.H.); seiki.shirai@shionogi.co.jp (S.S.); akiho.sakai@shionogi.co.jp (A.S.); takayuki.nakagawa@shionogi.co.jp (T.N.); kumiko.goto@shionogi.co.jp (K.G.); osamu.yoshida@shionogi.co.jp (O.Y.); kazufumi.katayama@shionogi.co.jp (K.K.); 3 Department of Biostatistics, Epidemiology and Informatics, Perelman School of Medicine, University of Pennsylvania, Philadelphia, PA 19104-6073, USA; alisaste@pennmedicine.upenn.edu (A.J.S.); ghchoi@pennmedicine.upenn.edu (G.C.); 4 Department of Basic and Translational Sciences, School of Dental Medicine, University of Pennsylvania, Philadelphia, PA 19104-6073, USA; ghc@upenn.edu

**Keywords:** therapeutic vaccine, herpes simplex virus type 2, glycoprotein D, adjuvant, genital herpes, HSV-2 DNA shedding, guinea pigs, mice, neutralizing antibodies, T cells

## Abstract

Herpes simplex virus type 2 (HSV-2) is a leading cause of genital ulcer disease and a major risk factor for acquisition and transmission of HIV. Frequent recurrent genital lesions and concerns about transmitting infection to intimate partners affect the quality of life of infected individuals. Therapeutic vaccines are urgently needed to reduce the frequency of genital lesions and transmission. S-540956 is a novel vaccine adjuvant that contains CpG oligonucleotide ODN2006 annealed to its complementary sequence and conjugated to a lipid that targets the adjuvant to lymph nodes. Our primary goal was to compare S-540956 administered with HSV-2 glycoprotein D (gD2) with no treatment in a guinea pig model of recurrent genital herpes (studies 1 and 2). Our secondary goals were to compare S-540956 with oligonucleotide ODN2006 (study1) or glucopyranosyl lipid A in a stable oil-in-water nano-emulsion (GLA-SE) (study 2). gD2/S-540956 reduced the number of days with recurrent genital lesions by 56%, vaginal shedding of HSV-2 DNA by 49%, and both combined by 54% compared to PBS, and was more efficacious than the two other adjuvants. Our results indicate that S-540956 has great potential as an adjuvant for a therapeutic vaccine for genital herpes, and merits further evaluation with the addition of potent T cell immunogens.

## 1. Introduction

Globally, 492 million people of ages 15 to 49 years are estimated to have a genital herpes infection caused by HSV-2, and 140 million by HSV-1 [1,2]. Incident (new) HSV-2 infections occurred in an estimated 572,000 people ages 15 to 49 years in the US in 2018, placing genital herpes as the fifth most common newly acquired sexually transmitted infection behind human papillomavirus, trichomonas, chlamydia, and gonorrhea [3]. HSV-2 infections persist for life, which accounts for the high prevalence of 18.6 million people ages 15 to 49 years with an HSV-2 infection, ranking second to HPV in terms of the prevalence of sexually transmitted infections [3]. People with genital herpes are often unaware of their infection status, but for the approximate 15% that are aware, anxiety about transmitting the infection to intimate partners is perhaps their biggest concern, while others worry about frequent, painful recurrent genital lesions [4,5]. Transmission of herpes from a mother to a newborn infant is a dreaded complication that occurs in 14,000 live births globally [6]. Genital herpes increases the risk of acquiring or transmitting HIV by three- to fourfold, and is helping to fuel the HIV pandemic [7]. These observations support the importance of developing vaccines for preventing genital herpes and for treating people who are already infected.

No vaccine has been approved by the US Food and Drug Administration (FDA) for preventing or treating genital herpes. Three publications have described large phase 3 human vaccine trials aimed at preventing genital herpes [8,9,10]. Each assessed one or more viral entry molecules. In one study, subjects were immunized with HSV-2 glycoproteins B (gB2) and D (gD2) with MF59^®^ as the adjuvant, and in the two other studies, individuals were immunized with gD2 alone, with monophosphoryl lipid A (MPL) and alum as adjuvants. No study achieved its primary endpoint of preventing genital lesions or subclinical genital infection, as measured by seroconversion to antigens not included in the vaccine.

The goals of a therapeutic vaccine are to reduce the frequency of recurrent genital lesions and/or genital shedding of HSV-2 DNA. Genital shedding indicates HSV reactivation and is a useful biomarker for the risk of transmission to intimate partners [11]. In 2019, a study supported by Genocea Biosciences reported a phase 2 human trial of a therapeutic vaccine, comparing a placebo with gD2 administered with HSV-2 infected cell polypeptide 4 (ICP4) and Matrix-M2^TM^ (MM2) adjuvant. The investigators noted a reduction in genital shedding of HSV-2 DNA over 71 days, but the response did not last beyond 6 months, while the vaccine reduced genital lesions by 50% over a 1-year period. The results suggest that shedding may be more difficult to control than lesions [12]. A decision was made not to pursue further human trials with the gD2/ICP4/MM2 vaccine, perhaps because the results were inferior to those reported for daily suppressive antiviral therapy with acyclovir or valacyclovir [13]. In subjects with up to 10 recurrent genital lesions annually, daily suppressive valacyclovir reduced the number of days with recurrent genital lesions and the genital shedding of HSV-2 DNA by 73%, although 40% of subjects still had at least 1 genital lesion and 50% had at least 1 day of HSV-2 DNA shedding over eight months [13]. A reasonable goal for a therapeutic vaccine is to outperform daily suppressive valacyclovir therapy when used alone, or, perhaps, in combination with valacyclovir.

Achieving the goal of outperforming acyclovir/valacyclovir will likely require the selection of immunogens and adjuvants that stimulate potent T cell responses [14,15,16,17,18,19]. Herein, we describe immune responses and outcomes using a novel adjuvant, S-540956, developed by Shionogi & Co., Ltd. (Osaka, Japan). S-540956 has a unique CpG oligonucleotide annealed to a complementary strand containing an amphiphilic chain unit. S-540956 acts as a potent cancer vaccine adjuvant by targeting draining lymph nodes [20]. We assessed the potency of S-540956 as a component of a therapeutic vaccine for genital herpes using a guinea pig genital herpes model [21,22]. We administered S-540956 with HSV-2 gD2 subunit protein to guinea pigs previously infected intravaginally with HSV-2, and evaluated the recurrent genital lesions and vaginal shedding of HSV-2 DNA. Our choice of gD2 alone was not intended to represent our future candidate vaccine for human trials; rather, we intended to determine whether this novel adjuvant had value as one component of a therapeutic vaccine for genital herpes. We compared the immune responses and vaccine efficacy of gD2 combined with S-540956 with subjects who received no treatment (PBS). We also evaluated gD2 administered with CpG (ODN2006) or glucopyranosyl lipid A formulated in a stable oil-in-water nano-emulsion (GLA-SE). Our results indicate that S-540956 is a potent adjuvant, worth pursuing as a component of a multi-antigen therapeutic vaccine for genital herpes.

## 2. Materials and Methods

### 2.1. Antigen and Adjuvants

The baculovirus gD2 antigen, bac-gD2(306t), is prepared in insect cells and has a purity > 95%. The gD2 antigen is truncated prior to the transmembrane domain and contains amino acids 26 to 331 of HSV-2 strain 333, where amino acid 26 is the first amino acid after the signal peptide [23,24]. Adjuvant S-540956 is a double-stranded oligodeoxynucleotide conjugated to a lipid and prepared from CpG ODN2006 (5′TCGTCGTTTTGTCGTTTTG TCGTT-3′) [20]. The complementary strand with the lipid ligand is referred to as C-540956. Alexa Fluor 647-labeled ODN2006 (ODN2006-AF647) was synthesized by Ajinomoto-Biopharma Services. Alexa Fluor 647-labeled S-540956 (S-540956-AF647) was composed of ODN2006 AF647 hybridized with C-540956. GLA-SE was purchased from Creative Biolabs (Shirley, NY, USA). Purified gD2 was formulated with S-540956 or GLA-SE according to the manufacturer’s instructions.

### 2.2. Animals

Female Hartley strain guinea pigs weighing 250–350 g were purchased from Charles River, Cambridge, MA, USA, for studies conducted at the University of Pennsylvania. Five-week-old female Hartley strain guinea pigs (SLC Inc., Hamamatsu, Japan) and six- to eight-week-old female C57BL/6J mice (CLEA, Higashiyama, Japan) were purchased for studies conducted at Shionogi & Co., Ltd. All animal experiments were conducted following the appropriate guidelines and with the approval of the University of Pennsylvania Institutional Animal Care and Use Committee (IACUC protocol 805187) or the Shionogi Animal Care and Use Committee (S19016C-0201).

#### 2.2.1. Immunology Assays Using Naïve Mice or Guinea Pigs Performed at Shionogi & Co., Ltd.

(a) Lymph node targeting of adjuvants in naïve guinea pigs. Animals were injected intramuscularly (IM) in a hind limb thigh muscle once with 9.42 nmole of S-540956-AF647 or ODN2006-AF647. Draining lymph nodes were collected 24 h later and analyzed using the IVIS imaging system (Perkin Elmer, Waltham, MA, USA) [20].

(b) Serum gD2 ELISA titers in naïve mice and guinea pigs. Serum gD2 total IgG, IgG1, or IgG2c ELISA was measured in 384-well plates coated with 1 μg/mL gD2 antigen overnight at 4 °C, blocked for 1 h with PBS containing 1% bovine serum albumin (Sigma-Aldrich, St. Louis, MI, USA), and incubated with serially diluted sera for 2 h. Horseradish peroxidase-conjugated anti-mouse or anti-guinea pig total IgG, anti-mouse IgG1, or IgG2c (Bethyl Laboratories, Inc., Montgomery, TX, USA) was added for 1 h, followed by 1-Step Ultra TMB-ELISA solution (Thermo Fisher Scientific, Waltham, MA, USA). The reaction was stopped after 5 min by the addition of 1 M sulfuric acid (FUJIFILM Wako Pure Chemical Co., Ltd., Osaka, Japan), and the optical density was measured at 450 nm (OD_450_) using EnVision 2103 (Perkin Elmer) [20].

(c) Neutralizing antibody titers in naïve guinea pig serum. Serum-neutralizing antibody titers were measured by a reporter cell line that produced luciferase in an HSV-2 inoculum dose-dependent manner. A truncated UL29 promoter fragment (582 bp upstream of ATG start codon) from the HSV-2 G strain was cloned into a KpnI/XhoI site of the luciferase vector pGL4.14 (Promega, Madison, WI, USA). The plasmid was transfected into HEp-2 cells (American Type Culture Collection, Manassas, VI, USA), and a stable cell line was selected that correlated the inoculum dose of HSV-2 with luciferase activity. To measure 50% neutralizing antibody titers (NT_50_), guinea pig serum was serially diluted and incubated with HSV-2 strain G (1.2 × 10^3^ PFU) in 96-well plates for 1 h at 37 °C and 5% CO_2_. The reporter cells (3 × 10^4^ cells) were added for 2 days at 37 °C with 5% CO_2_; then, ONE-Glo^™^ Luciferase Assay System (Promega) was added to each well, and the intensity of luminescence was measured using EnVision 2103 (Perkin Elmer). Nonlinear regression was used to determine NT_50_.

(d) Flow cytometry in naïve guinea pigs. To analyze gD2-specific CD8^+^ T cell responses, splenocytes from immunized guinea pigs were labeled using a Cell Trace™ Violet (CTV) Cell Proliferation Kit (Thermo Fisher Scientific) according to the manufacturer’s protocol and stimulated with 10 μg/mL gD2 antigens or 10 μg/mL concanavalin A (Sigma-Aldrich) for 5 days at 37 °C in 5% CO_2_. The harvested cells were stimulated with 50 ng/mL phorbol 12-myristate 13-acetate (PMA, Sigma-Aldrich) and 10 μM ionomycin calcium (Sigma-Aldrich) in the presence of 1 μg/mL brefeldin A (Thermo Fisher Scientific) for 4 h at 37 °C in 5% CO_2_. For intracellular cytokine-staining assays, cells were fixed with 4% paraformaldehyde phosphate buffer solution (FUJIFILM Wako Pure Chemical Co., Ltd.) for 10 min at room temperature, followed by a permeabilization buffer (Thermo Fisher Scientific) on ice. Dead cells were detected using a Zombie NIR™ Fixable Viability Kit (Biolegend, San Diego, CA, USA). CD8^+^IFNγ^+^ T cells were detected using FITC-conjugated anti-guinea pig CD8 (clone CT6, Bio-Rad Laboratories, Hercules, CA, USA), PE-conjugated anti-guinea pig CD4 (clone CT7, GeneTex, Irvine, CA, USA), and PF647P-conjugated anti-guinea pig IFNγ (clone MT4A27, Mabtech, Mariemont, OH, USA), which were added for 45 min on ice. Data were collected and analyzed using the Novocyte flow cytometer system (Agilent Technologies, Santa Clara, CA, USA). Proliferated CD8^+^IFNγ^+^ cells were defined as gD2-specific CD8^+^IFNγ^+^ T cells.

(e) Antibody-dependent cellular cytotoxicity (ADCC) reporter assay in naïve mice. The assay was modified from that previously reported in [25]. To generate target cells, the gD2 open reading frame from the HSV-2 G strain was cloned into pCDNA3.1 by GenScript (Piscataway, NJ, USA). HEK293 cells (3 × 10^6^ cells) were transfected with the gD2 plasmid (15 μg) and incubated for 24 h at 37 °C with 5% CO_2_. The target cells were detached and incubated in 96-well plates (1.5 × 10^4^ cells) for 24 h at 37 °C with 5% CO_2_. Diluted sera (1:50 dilution) and FcγRIV reporter cells (7.5 × 10^4^ cells) (Mouse FcγRIV ADCC Bioassay, Complete Kit, Promega) were added for 24 h at 37 °C with 5% CO_2_ [26]. FcγRIV activation was determined by measuring the intensity of luminescence with a microplate reader, EnVision 2103 (Perkin Elmer).

(f) ELISPOT assay in naïve mice. gD2-spcific T cell responses of murine splenocytes were measured using a Mouse IFN-γ ELISpot Plus kit (ALP) (Mabtech AB) according to the manufacturer’s protocol. Splenocytes (8.0 × 10^5^ cells/well) were incubated with 2 μg/mL gD2 for 20–24 h at 37 °C with 5% CO_2_. The number of spots was counted using an ImmunoSpot S6 MICRO analyzer (Cellular Technology Limited LLC, Beachwood, OH, USA).

#### 2.2.2. Immunology Assays in HSV-2 Infected Guinea Pigs Performed at the University of Pennsylvania

(a) Serum and vaginal ELISA. Serial two-fold dilutions of serum or vaginal fluids were used to measure gD2 endpoint titers [27].

(b) Serum-neutralizing antibody titers. Serial two-fold dilutions of serum were incubated with 100 PFU of HSV-2 strain MS for 1 h at 37 °C, with 5% human serum from an HSV-1 and HSV-2 negative donor as the source of the complement. Then, they were added to a Vero cell monolayer for two to three days under a methylcellulose overlay prior to counting plaques. The endpoint titer was the serum dilution that reduced the plaque number by ≥50% [28].

(c) HSV-2 DNA copy number in vaginal swabs. Vaginal swabs were obtained on multiple days, starting one day after the first immunization, and placed in Dulbecco’s modified Eagle’s medium (DMEM, containing HEPES, L-glutamine, and antibiotics) and 5% fetal bovine serum. DNA was purified from 200 μL (QiaCube HT), and 5 μL of purified DNA was amplified (Roche LightCycler 96) using HSV-2 U_s_9 DNA primers and a probe [29]. Samples with less than 1 copy of HSV-2 DNA by 40 cycles were considered negative. Positive samples were confirmed in duplicate. The assay limit of detection was 200 copies of HSV-2 DNA/mL.

#### 2.2.3. Therapeutic Vaccine Studies in Guinea Pigs at the University of Pennsylvania

Female guinea pigs were infected intravaginally with 2 × 10^5^ plaque-forming units of HSV-2 strain MS that had been passaged in a cell culture to attenuate lethality [21]. Animals were randomized into equal groups based on the number of days with genital lesions for the first 14 days post-infection. Scoring for genital lesions was performed blinded to the vaccine groups after the first immunization by two investigators. A score of 0 was assigned for no genital lesions, and 1 for one or more genital lesions. The score was based on a consensus between the two investigators. After two weeks, animals seldom had more than one lesion, which often resolved within one or two days. Vaginal swabs were obtained for HSV-2 DNA by qPCR for four to five days weekly after the first immunization [30,31]. Vaginal fluids were collected using an eye spear swab (BVI) that was placed in 100 μL PBS and centrifuged to elute antibodies prior to being discarded [27].

### 2.3. Statistical Analysis

The primary analysis compared gD2/S-540956 with PBS. Other comparisons were considered secondary. Details of the statistical methods are noted in the figure legends. GraphPad Prism, version 9.4.1, was used to prepare graphs and for statistical analysis. A *p* value of <0.05 was considered significant.

## 3. Results

### 3.1. Study 1: S-540956 Administered with gD2 Compared to ODN2006 Administered with gD2, gD2 Alone, or PBS

The goal of study 1 was to determine whether S-540956 is worth pursuing as an adjuvant in a therapeutic vaccine for genital herpes. We performed studies in mice and guinea pigs to assess immunogenicity and vaccine efficacy by (i) comparing gD2/S-540956 with no treatment (PBS); and (ii) comparing gD2/S-540956 with gD2 alone or gD2 administered with ODN2006 (gD2/ODN2006), where ODN2006 is a CpG adjuvant that is a component of S-540956.

#### 3.1.1. Lymph Node Trafficking and Immunology Studies in Naïve Guinea Pigs

**Figure 1:**
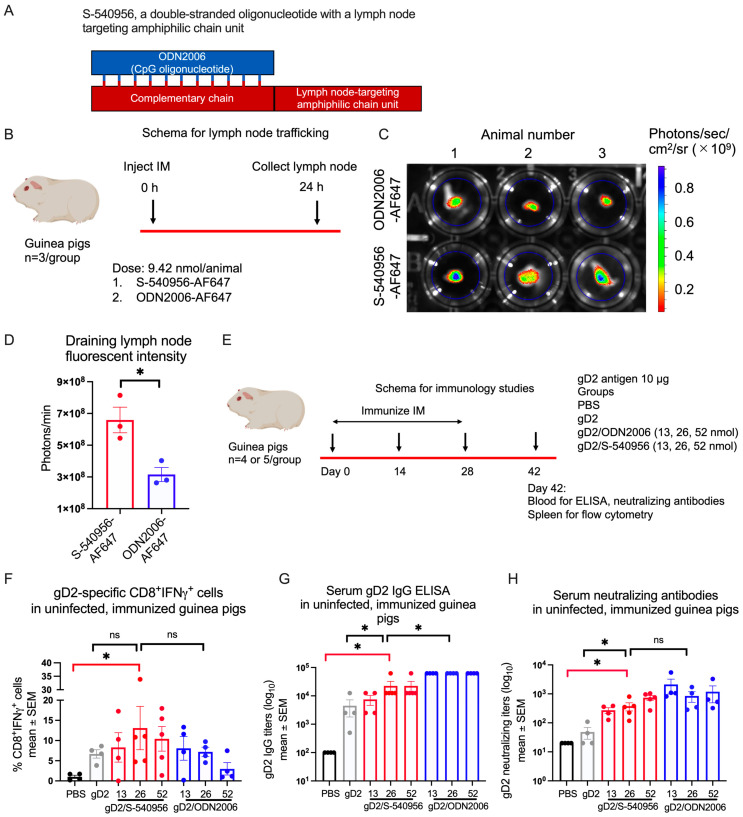
Evaluating S-540956 and ODN2006 adjuvants in guinea pigs. (**A**) Adjuvant design for ODN2006 and S-540956. The single-stranded ODN2006 is shown in blue. S-540956 is double-stranded, consisting of ODN2006 and a complementary strand, shown in red, that is linked to an amphiphilic chain. (**B**) Experimental design to detect trafficking of ODN2006-AF647 or S-540956-AF647 to draining lymph nodes in naïve guinea pigs. (**C**,**D**) Draining lymph nodes were collected at 24 h, and fluorescence intensity was recorded as mean photons per draining lymph node (n = 3/group). The *p* value was calculated by unpaired *t*-test with Welch’s correction. (**E**) Experimental design for immunizing naïve guinea pigs with PBS, gD2 alone, or gD2 administered with 13, 26, or 52 nmol ODN2006 or S-540956. (**F**–**H**) Splenocyte CD8^+^IFNγ^+^ responses (% CD8^+^IFNγ^+^CTV^−^ cells), serum gD2 IgG titers, and serum-neutralizing antibody titers were measured on day 42. *p* values in (**F**–**H**) were calculated by the Mann–Whitney test, with the primary comparison between gD2/S-540956 and PBS (highlighted by red brackets) and with no adjustment for multiple comparisons. n = 4 per group, except gD2/S-540956 at 26 or 52 nmol, n = 5/group. * *p* < 0.05; ns: *p* value not significant.

Adjuvant S-540956 consists of CpG oligonucleotide ODN2006, which is annealed to its complementary strand and has an amphiphilic chain unit that enhances binding to albumin in order to target draining lymph nodes (Figure 1A) [20]. Alexa Fluor 647 (AF647) dye was conjugated to S-540956 or ODN2006 and injected IM into guinea pigs to evaluate trafficking to the regional draining lymph nodes (schema Figure 1B). The accumulation of fluorescent signals was measured at 24 h (Figure 1C). The accumulation of S-540956-AF647 was twofold higher than ODN2006-AF647 (Figure 1D), indicating that S-540956 targets lymph nodes in guinea pigs, as we previously reported in mice [20].

We investigated whether S-540956 would enhance immune responses in naïve (uninfected) guinea pigs when administered with gD2, a glycoprotein that has been widely used in human vaccine trials [8,9,10]. Naïve guinea pigs were immunized with PBS; 10 μg gD2 alone; or gD2 administered with 13, 26, or 52 nmol of S-540956 or ODN2006 (see schema Figure 1E). We evaluated multiple concentrations of S-540956 and ODN2006 to aid in selecting an optimal dose to use in subsequent studies on HSV-2-infected guinea pigs. S-540956 induced the most potent gD2-specific CD8^+^IFNγ^+^ response at a concentration of 26 nmol, which was significantly higher than in the PBS control (Figure 1F and Figure S1), and despite being greater than gD2 alone or gD2 with 26 nmol ODN2006, those differences did not reach statistical significance (Figure 1F). At a concentration of 26 nmol, gD2/S-540956 induced gD2 IgG ELISA titers that were significantly higher than the PBS or gD2 only groups, but not as high as gD2 with 26 nmol ODN2006 (Figure 1G). Neutralizing antibody titers were higher when gD2 was administered with 26 nmol S-540956 than PBS or gD2 alone, but no different than when gD2 was administered with 26 nmol ODN2006 (Figure 1H). We conclude that at 26 nmol, gD2/S-540956 stimulates gD2-specific CD8^+^IFNγ^+^ T cell responses, IgG binding titers, and neutralizing antibody titers in naïve guinea pigs.

#### 3.1.2. Antibody Responses to Adjuvanted gD2 in HSV-2-Infected Guinea Pigs

**Figure 2:**
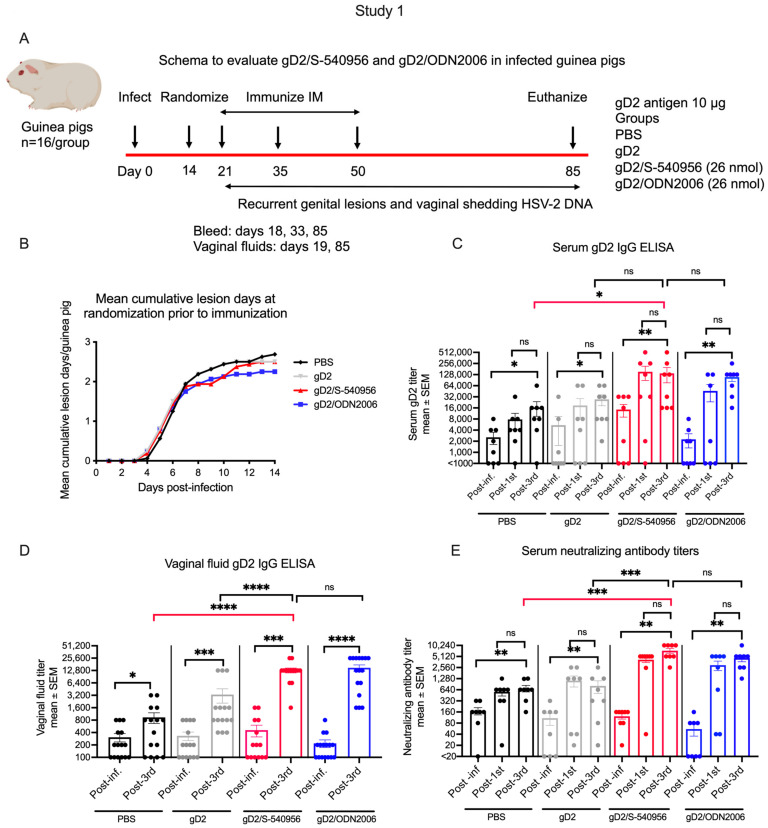
Antibody responses in HSV-2-infected guinea pigs immunized with PBS, gD2 alone, or gD2 administered with 26 nmol ODN2006 or S-540956. (**A**) Experimental design of studies in infected and subsequently immunized animals. (**B**) Mean number of days with genital lesions prior to randomization on day 14 (n = 16/group). (**C**) Serum gD2 IgG ELISA titers (n = 8/group). (**D**) Vaginal fluid gD2 IgG ELISA titers (n = 15–16/group). (**E**) Serum-neutralizing antibody titers in the presence of human complement (n = 8/group). *p* values in (**C**–**E**) were calculated using the Wilcoxon matched-pairs signed rank test for within-group comparisons, and the two-tailed Mann–Whitney test for cross-group comparisons. The primary analyses between gD2/S-540956 and PBS are highlighted by red brackets. * *p* < 0.05; ** *p* < 0.01; *** *p* < 0.001; **** *p* < 0.0001; ns: *p* value not significant.

We next used the guinea pig therapeutic vaccine model of genital herpes to evaluate the antibody responses of serum and vaginal fluid to immunization. In this model, guinea pigs were first infected intravaginally with HSV-2, allowed to recover, and then immunized with the vaccine candidates (schema Figure 2A) [32]. Sixty-four Hartley strain female guinea pigs were infected intravaginally with HSV-2 and randomized into four groups based on the number of days with genital lesions prior to immunization. At the time of randomization, on day 14, all four groups had a similar number of days with genital disease, indicating that the randomization of animals into each immunization groups was unbiased (Figure 2B). The primary comparison for these studies was between gD2/S-540956 and PBS. In the PBS group, serum gD2 IgG ELISA titers, vaginal gD2 IgG ELISA titers, and serum-neutralizing antibody titers were each elevated when comparing the post-infection period with the end of the study, indicating the impact of HSV-2 recurrence on stimulating antibody titers in the absence of immunization (Figure 2C–E). However, antibody titers were significantly higher at the end of the study (post-3rd immunization) in the gD2/S-540956 group than the PBS group (Figure 2C–E), while vaginal fluid and neutralizing antibody titers were also significantly higher in the gD2/S-540956 group than the gD2 only group (Figure 2D,E). Neither serum nor vaginal gD2 IgG titers, nor serum-neutralizing antibody titers, differed significantly when comparing the gD2/S-540956 group with the gD2/ODN2006 group (Figure 2C–E). Serum gD2 IgG titers and serum-neutralizing antibody titers changed little when comparing the post-first and post-third immunization periods for all groups (Figure 2C,E). We conclude that the immunization of previously infected guinea pigs boosted antibody titers in the gD2/S-540956 group predominantly after the first immunization, and that gD2/S-540956 outperformed the PBS group.

#### 3.1.3. Effects of Immunization on Recurrent Genital Herpes Lesions and Vaginal Shedding of HSV-2 DNA in HSV-2-Infected Guinea Pigs

**Figure 3:**
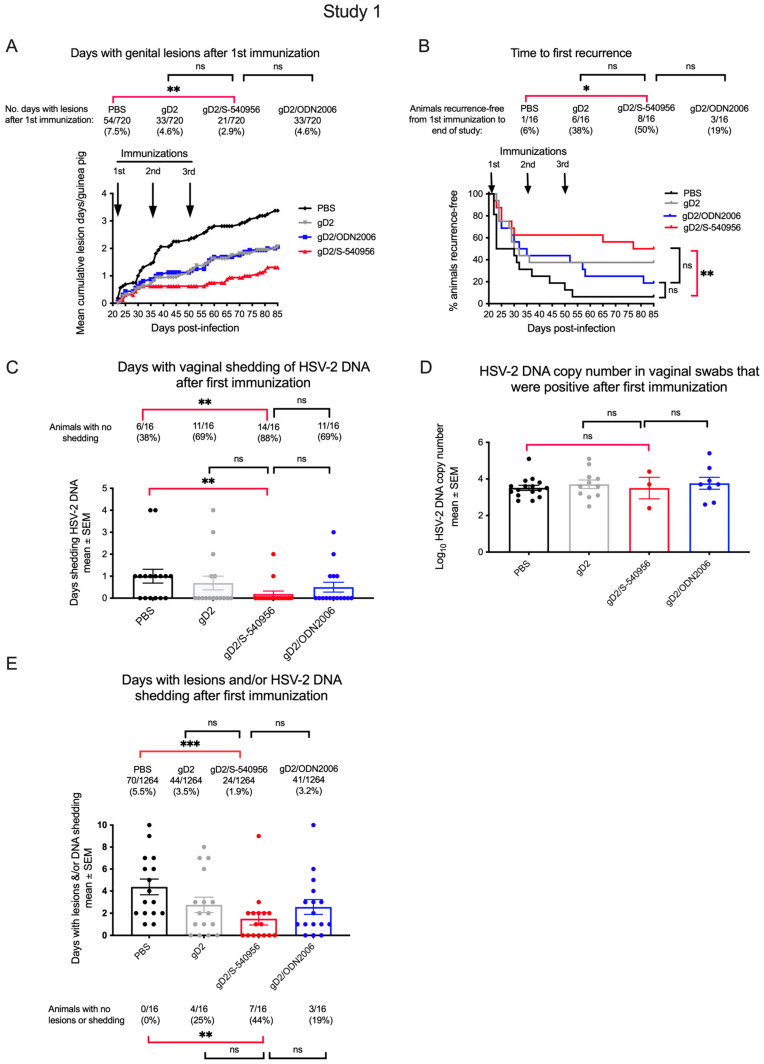
Vaccine efficacy in HSV-2 infected guinea pigs immunized with PBS, gD2 alone, or gD2 administered with 26 nmol ODN2006 or S-540956. (**A**) Lesion days, from one day after the first immunization until the end of the study. *p* values comparing curves are presented above the curves; these were determined by the two-tailed Mann–Whitney test without adjustment for multiple comparisons. (**B**) Time to first recurrence after the first immunization. *p* values were calculated by the log-rank test. *p* values for the number of animals that were recurrence-free (above the graph) were calculated by the two-tailed Fisher’s exact test. (**C**) Days with vaginal shedding of HSV-2 DNA. *p* values for the figure were calculated by the two-tailed Mann–Whitney test, while *p* values for the number of animals with no shedding (above the graph) were calculated by the two-tailed Fisher’s exact test. (**D**) HSV-2 DNA copy number on days with vaginal shedding after the first immunization. *p* values were calculated by the two-tailed Mann–Whitney test. (**E**) Combined days after the first immunization were calculated as the sum of days with recurrent genital lesions and with recurrent vaginal shedding of HSV-2 DNA. *p* values for the total days with lesions and shedding (above the graph) were calculated by the two-tailed Mann–Whitney test, while the number of animals that had no lesions or shedding (below the graph) was calculated by the two-tailed Fisher’s exact test. n = 16/group. The primary analyses between gD2/S-540956 and PBS are highlighted by red brackets. * *p* < 0.05; ** *p* < 0.01; *** *p* < 0.001; ns: *p* value not significant.

We next evaluated the efficacy of the various therapeutic vaccine candidates by assessing the number of days for which animals had recurrent genital lesions when immunized with PBS, gD2 alone, gD2/S-540956, or gD2/ODN2006. Scoring began 1 day after the first immunization, resulting in 720 observation days for each group (n = 16/group). The gD2/S-540956 group had significantly fewer recurrent lesion days than the PBS group (Figure 3A). The data above the graph show the percentage of the days during the study period for which animals in each group had genital lesions (Figure 3A). The gD2/S-540956 group also had a delayed time to first recurrence compared to the PBS group (Figure 3B). The percentage of animals that were recurrence-free at the end of the study is shown above the graph (Figure 3B).

Vaginal shedding of HSV-2 DNA in guinea pigs is a marker for the reactivation of a virus from latency [33]. Vaginal swabs were evaluated for genital shedding of HSV-2 DNA on 544 days for each group, beginning after the first immunization. Animals in the gD2/S-540956 group had significantly fewer days of HSV-2 DNA shedding than the PBS group (Figure 3C). The gD2/S-540956 group also outperformed the gD2 only and gD2/ODN2006 groups, but these differences did not achieve statistical significance (Figure 3C). The number of animals with no shedding was significantly higher in the gD2/S-540956 than the PBS group (data above graph Figure 3C). Although the gD2/S-540956 group had fewer shedding days than other groups, the HSV-2 DNA copy number did not differ among groups on days on which animals had shedding (Figure 3D).

Fewer days with genital lesions and HSV-2 DNA shedding are both important endpoints for a therapeutic genital herpes vaccine. We calculated the number of days for which each animal had genital lesions and/or vaginal shedding of HSV-2 DNA (Figure 3E). The total number of days with lesions and/or shedding for each group is shown above the graph, while the number of animals with no lesions or HSV-2 DNA shedding is shown below the graph. The gD2/S-540956 group had significantly fewer days of genital lesions and/or HSV-2 DNA shedding than the PBS group, while the differences between the gD2/S-540956, gD2 only, and gD2/ODN2006 groups were not statistically significant (Figure 3E). We conclude that the gD2/S-540956 group significantly outperformed the PBS group in terms of days with genital lesions, time to first recurrence, days with vaginal shedding of HSV-2 DNA, and combined days with lesions and/or shedding.

### 3.2. Study 2: S-540956 Compared to GLA-SE or PBS

The primary objective of study 2 was to repeat the results of study 1, which demonstrated that gD2/S-540956 outperformed the PBS group. A second objective was to compare S-540956, a TLR9 agonist, with GLA-SE, a TLR4 agonist [34]. We chose GLA-SE in part because, at the time we designed the study, a clinical trial was using GLA-SE as an adjuvant (ClinicalTrials.gov identifier: NCT04222985). That trial has since been discontinued.

#### 3.2.1. Immunology Studies in Naïve Mice and Guinea Pigs

**Figure 4:**
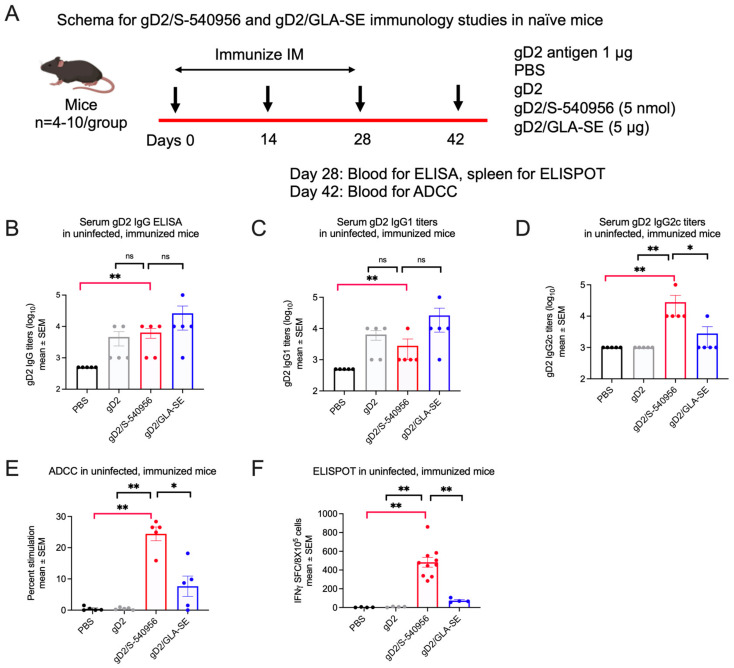
Antibody and cellular immune responses in uninfected (naïve) mice immunized with PBS, gD2 alone, gD2/S-540956, or gD2/GLA-SE. (**A**) Experimental design for immunization studies in uninfected mice. (**B**–**E**) Serum gD2 IgG ELISA, serum gD2 IgG1 ELISA, serum gD2 IgG2c ELISA, and serum ADCC titers (n = 5/group). (**F**) ELISPOT to detect IFNγ^+^ T cells in splenocytes (n = 4/group except n = 10 in gD2/S-540956). *p* values were calculated by the two-tailed Mann–Whitney test, with the primary comparison between gD2/S-540956 and PBS (highlighted by red brackets). * *p* < 0.05; ** *p* < 0.01; ns: *p* value not significant.

Naïve mice: Analytical tools for ADCC and T cell studies are limited in guinea pigs. Therefore, we first compared the immune responses to S-540956 and GLA-SE in mice (schema Figure 4A). Serum gD2 IgG ELISA titers were significantly higher in the gD2/S-540956 mice than in the PBS group (Figure 4B), with a predominant IgG2c isotype response, suggesting a T helper type 1 (Th1) response (Figure 4C,D). Antibody-dependent cellular cytotoxicity (ADCC) and T cell enzyme-linked immunospot (ELISPOT) responses were also significantly higher in the gD2/S-540956 mice than the PBS controls or the gD2 only or gD2/GLA-SE groups (Figure 4E,F).

**Figure 5:**
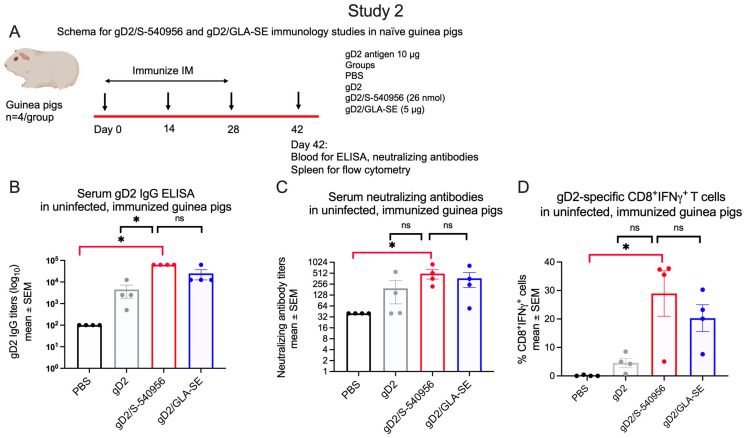
Antibody and cellular immune responses in uninfected (naïve) guinea pigs immunized with PBS, gD2 alone, gD2/S-540956, or gD2/GLA-SE. (**A**) Experimental design for immunization studies in uninfected guinea pigs. (**B**–**D**) Serum gD2 IgG ELISA, serum-neutralizing antibody titers, and gD2-specific CD8^+^IFNγ^+^ T cells (n = 4/group). *p* values were calculated by the two-tailed Mann–Whitney test, with the primary comparison between gD2/S-540956 and PBS (highlighted by red brackets). * *p* < 0.05; ns: *p* value not significant.

Naïve guinea pigs: We next evaluated the antibody and CD8^+^IFNγ^+^ T cell responses in uninfected guinea pigs. Animals were immunized three times at two-week intervals with gD2/S-540956, gD2/GLA-SE, gD2 alone, or PBS. Serum and splenocytes were obtained two weeks after the final immunization (schema: Figure 5A). Serum gD2 IgG ELISA titers and neutralizing antibody titers were significantly higher in the gD2/S-540956 group than in the PBS group, as were gD2-specific CD8^+^IFNγ^+^ T cell responses (Figure 5B–D). gD2/S-540956 also outperformed the gD2 alone and gD2/GLA-SE groups, although only some differences achieved statistical significance (Figure 5B–D). The results were consistent in studies 1 and 2, which compared gD2/S-540956 with PBS or gD2 alone for gD2 IgG ELISA, neutralizing antibodies, and gD2-specific CD8^+^IFNγ^+^ T cell responses in naïve guinea pigs (Figure 1F–H and Figure 5B–D). In both studies, gD2 IgG ELISA, neutralizing antibodies, and T cell responses were significantly higher in gD2/S-540956 animals than in the PBS controls. Where differences were noted comparing gD2/S-540956 to gD2 alone, the responses favored gD2/S-540956.

#### 3.2.2. Antibody Responses to gD2/S-540956, gD2/GLA-SE, or PBS Immunization in Previously HSV-2-Infected Guinea Pigs

**Figure 6:**
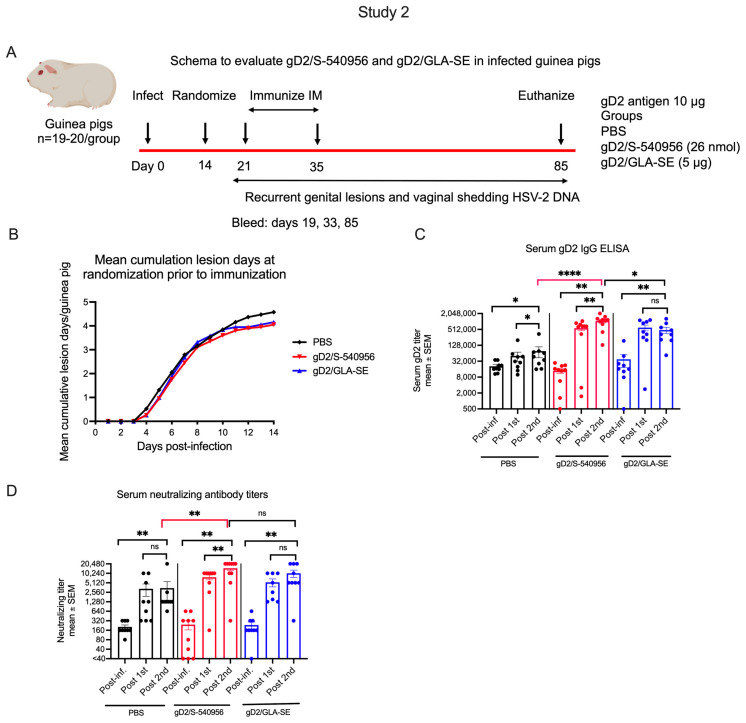
Antibody responses in infected guinea pigs immunized with PBS, gD2/S-540956, or gD2/GLA-SE. (**A**) Experimental design of studies in infected and subsequently immunized animals. (**B**). Mean cumulative days with genital lesions prior to randomization on day 14 (n = 19 in PBS and gD2/GLA-SE, n = 20 in gD2/S-540956). (**C**,**D**) Serum gD2 IgG ELISA titers and neutralizing antibody titers in the presence of complements (n = 8–10/group). *p* values in (**C**,**D**) were calculated by the Wilcoxon matched-pairs signed rank test for comparisons within groups, while comparisons between groups were performed by the two-tailed Mann–Whitney test. * *p <* 0.05; ** *p <* 0.01; **** *p <* 0.0001; ns: *p* value not significant.

We then assessed serum antibody responses to immunogens in guinea pigs that were previously infected intravaginally with HSV-2 (schema Figure 6A). Animals were immunized twice, on days 21 and 35, in study 2 instead of 3 times, as in study 1. The decision to use two immunizations was based on observations in study 1 that the third immunization failed to boost gD2 IgG ELISA antibody titers or neutralizing antibody titers beyond those noted after the first immunization (Figure 2C,E), and the slope of the lesion curve did not flatten after the third immunization (Figure 3A).

Animals were randomized prior to immunization into three groups based on the number of days with lesions through day 14 (Figure 6B). Sera obtained post-infection, post-first immunization, and post-second immunization were evaluated for gD2 IgG ELISA titers and neutralizing antibody titers. As in study 1 (Figure 2C,E), infection significantly increased the serum gD2 IgG and serum-neutralizing antibody titers in the PBS group by the end of the experiment; however, the boost in titers was significantly greater in the gD2/S-540956 group (Figure 6C,D). The gD2/S-540956, but not the gD2/GLA-SE, group demonstrated a significant boost in gD2 IgG and neutralizing antibody titers when comparing the first and second immunizations (Figure 6C,D). We detected a significant difference comparing the post-second gD2 IgG ELISA titer of gD2/S-540956 with gD2/GLA-SE (Figure 6C). We conclude that the results of study 2 reproduced those of study 1 for the effects of immunization, boosting titers post-infection and detecting significant differences between the gD2/S-540956 and PBS groups for ELISA and neutralizing antibody titers. We detected a small, but statistically significant, boost when comparing the first and second immunization in the gD2/S-540956 group in study 2, which we did not observe when comparing the first and third immunization in study 1 (Figure 2C,E and Figure 6C,D).

#### 3.2.3. Effects of gD2/S-540956, gD2/GLA-SE, or PBS Immunization on Genital Lesions and Vaginal Shedding of HSV-2 DNA

**Figure 7:**
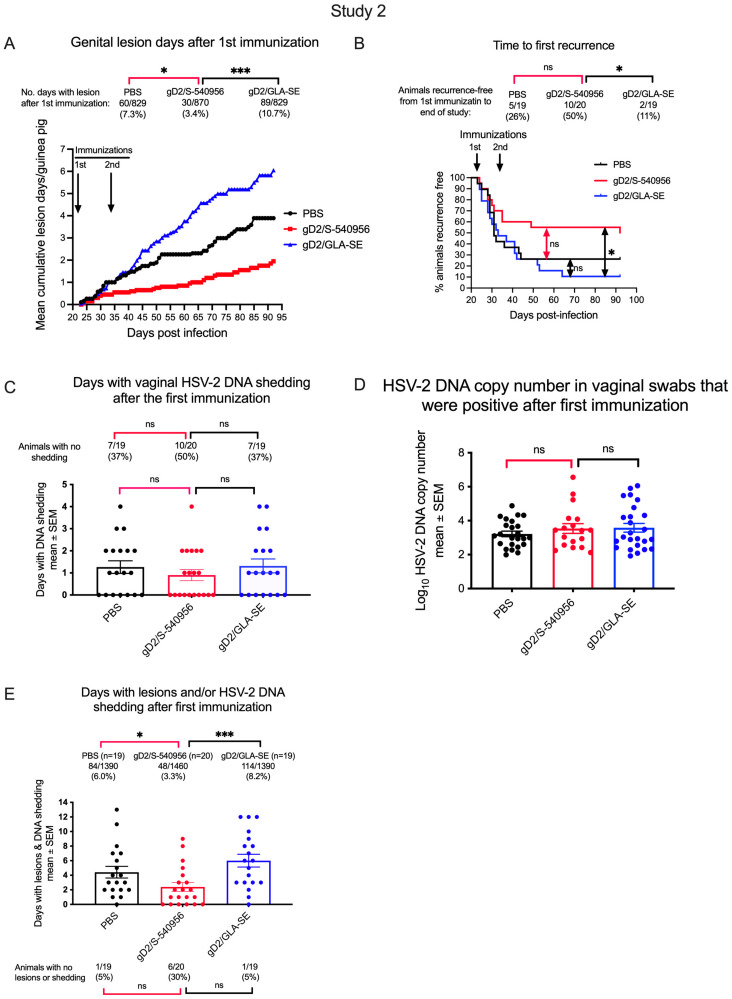
Vaccine efficacy in infected guinea pigs immunized with PBS, gD2/S-540956, or gD2/GLA-SE. (**A**) Mean cumulative days with recurrent genital lesions after the first immunization. Days with lesions for each group are shown above the graph. *p* values (unadjusted) were calculated by the Mann–Whitney test, according to the rate (count divided by number of observation days). (**B**) Time to first recurrence after the first immunization. *p* values were calculated by the log-rank test. (**C**) Days with vaginal HSV-2 DNA shedding after the first immunization. *p* values were calculated by the two-tailed Mann–Whitney test. *p* values for the number of animals without shedding were calculated by the two-tailed Fisher’s exact test (above graph). (**D**) HSV-2 DNA copy number on days with vaginal shedding after the first immunization. *p* values were calculated by the two-tailed Mann–Whitney test. (**E**) Combined days after the first immunization were calculated as the sum of days with genital lesions and vaginal shedding of HSV-2 DNA. *p* values for the total days with lesions and shedding (above the graph) were calculated as in Figure 7A. *p* values for the number of animals that had no lesions or shedding (below the graph) were calculated by the two-tailed Fisher’s exact test. n = 19 animals in the PBS and gD2/GLA-SE groups and 20 in the gD2/S-540956 group. The primary comparisons were between gD2/S-540956 and PBS (highlighted by red brackets). * *p <* 0.05; *** *p <* 0.001; ns: *p* value not significant.

Next, we evaluated the efficacy of gD2/S-540956, gD2/GLA-SE, and PBS (control) in reducing the number of days with genital herpes recurrence and/or days with recurrent vaginal shedding of HSV-2 DNA. The study began 1 day after the first immunization, for a total of 829 observation days for the PBS and gD2/GLA-SE groups (n = 19/group) and 870 days for the gD2/S-540956 group (n = 20/group). The gD2/S-540956 group had significantly fewer recurrent lesion days than the PBS, reproducing the results of study 1 (Figure 7A). Unexpectedly, the gD2/GLA-SE group had significantly more recurrent lesion days than the PBS group (Figure 7A). This surprising result may provide clues as to the type of immune response that is detrimental to controlling recurrent genital lesions, a subject for future investigation. Data above the graph display the results as percentages of the days during the study period for which each group had recurrent genital lesions (Figure 7A).

We evaluated the time to first recurrence. Differences between the gD2/S-540956 and PBS groups favored the gD2/S-540956 group, but did not achieve statistical significance, while the gD2/S-540956 group had a significantly delayed time to first recurrence compared to the gD2/GLA-SE group (Figure 7B). The percentage of animals in each group that was recurrence-free at the end of the study is shown above the graph (Figure 7B).

Vaginal swabs obtained after the first immunization were assessed for the number of days with vaginal shedding of HSV-2 DNA and HSV-2 DNA copy number. Swabs were collected on 561 days for the PBS or gD2/GLA-SE groups and on 590 days for the gD2/S-540956 group. The gD2/S-540956 group had fewer days with vaginal HSV-2 DNA shedding than the gD2/GLA-SE and PBS groups (Figure 7C), as well as more animals that never shed HSV-2 DNA (data above Figure 7C), although the differences were not statistically significant. On days with shedding, the HSV-2 DNA copy number did not differ among the groups (Figure 7D).

We calculated the combined days for which each animal had genital lesions and/or vaginal shedding of HSV-2 DNA (Figure 7E). The total number of days with lesions and/or shedding for each group is shown above the graph, while the number of animals with no lesions or shedding is shown below the graph. The gD2/S-540956 group had significantly fewer total days of lesions and/or HSV-2 DNA shedding than the PBS, reproducing the results of study 1 (Figure 7E). The gD2/S-540956 group also outperformed the gD2/GLA-SE group.

### 3.3. Combined Vaccine Efficacy Results from Studies 1 and 2

Our primary objective was to determine whether immunizing guinea pigs with gD2/S-540956 would reduce the number of days of recurrent genital lesions and vaginal shedding of HSV-2 DNA, compared with a control group that received PBS. We calculated the vaccine’s efficacy starting one day after the first immunization (Table 1). In both studies, gD2/S-540956 outperformed PBS in reducing days with recurrent genital lesions, recurrent HSV-2 DNA shedding, and combined days with genital lesions and/or HSV-2 DNA shedding. The vaccine efficacy of gD2/S-540956 for the combined studies 1 and 2 was 56.4% for reducing recurrent genital lesions, 48.8% for reducing recurrent days of vaginal shedding of HSV-2 DNA, and 54.4% for reducing days with combined lesions and/or shedding (Table 1).

We evaluated the number of animals that remained free of recurrent genital lesions and/or recurrent vaginal shedding of HSV-2 DNA in the gD2/S-540956 and PBS groups for individual studies 1 and 2, as well as the two studies combined. These data are based on the results of study 1 shown above Figure 3B,C, and those of study 2 above Figure 7B,C (Table 2). We conclude that significantly more animals in the gD2/S-540956 than PBS groups had no recurrent genital lesions and no vaginal shedding of HSV-2 DNA.

We evaluated the vaccine efficacy of gD2/S-540956 compared with PBS starting one day after the second immunization (Table S1) instead of one day after the first immunization (Table 1). The vaccine efficacy of gD2/S-54096 was 59.4% for reducing days with recurrent genital lesions, 37.5% for recurrent vaginal shedding of HSV-2 DNA, and 53.3% for combined days with lesions and/or shedding. We conclude that the efficacy of the gD2/S-540956 vaccine changed little whether analyzed after the first or second immunization, and that the efficacy of gD2/S-540956 was approximately 54% in terms of reducing days with genital lesions and vaginal shedding of HSV-2 DNA compared to PBS, whether evaluated after the first or second immunization.

We next compared the vaccine efficacy of gD2/S-540956 to that of gD2/ODN2006 and gD2/GLA-SE (Table 3). The combined efficacy for genital lesions and HSV-2 DNA shedding of gD2/S-540956 compared to gD2/ODN2006 was 41.4%, and compared to gD2/GLA-SE, it was 59.9%. We conclude that gD2/S-540956 outperformed these two other adjuvants when combined with the gD2 protein.

## 4. Discussion

We evaluated the immunogenicity and efficacy of a novel adjuvant, S-540956, administered with gD2 in murine and guinea pig models. Our primary analysis compared gD2/S-540956 with PBS (no treatment). Other comparisons of interest were between S-540956, ODN2006, and GLA-SE. These adjuvants were selected for comparison because ODN2006 is a CpG adjuvant that is a component of S-540956, and CpG is approved as an adjuvant in a hepatitis B vaccine by the FDA. GLA-SE is effective as an adjuvant when administered with three HSV-2 subunit protein antigens, gD2, UL19 (encodes a capsid protein), and UL25 (encodes capsid DNA packaging protein) in the guinea pig model of genital herpes [34,35]. In addition, clinical trials for multiple pathogens use GLA-SE as an adjuvant, including influenza, mycobacterium tuberculosis, schistosomiasis, and genital herpes, although the trial for genital herpes was recently terminated (ClinicalTrials.gov identifier: NCT04222985) [36,37,38]. Our primary endpoints were days with recurrent genital lesions and recurrent vaginal shedding of HSV-2 DNA in previously infected guinea pigs. Vaccine efficacy was assessed starting one day after the first immunization in animals receiving either three immunizations (study 1) or two immunizations (study 2). After the first immunization, the vaccine efficacy of the gD2/S-540956 immunogen for the two studies combined was 56% for genital lesions and 49% for vaginal shedding of HSV-2 DNA. After the second immunization, the vaccine efficacy was 59% for genital lesions and 38% for HSV-2 DNA shedding. These impressive results were achieved using only a single immunogen, gD2. Adding other immunogens, particularly immediate early, tegument, and/or capsid proteins that act as effective T cell antigens, may further enhance the efficacy of a therapeutic vaccine [14,15,16,17,18,19].

We compared the vaccine efficacy of S-540956 with ODN2006, another CpG adjuvant, but without the targeting amphiphilic chain unit on S-540956. gD2/S-540956 outperformed gD2/ODN2006 in terms of combined days with genital lesions and/or HSV-2 DNA shedding. The mechanisms that may have contributed to these results include: (i) CpG stimulates TLR9 on antigen-presenting cells to produce an inflammatory and anti-viral innate immune response [39,40]. The enhanced targeting of CpG adjuvant S-540956 to draining lymph nodes may have stimulated an increased production of type 1 interferons, which are important antiviral defenses. (ii) IgG ELISA and neutralizing antibody titers did not differ when these two adjuvants were compared in HSV-2 infected guinea pigs, suggesting that T cell responses, particularly gD2-specific CD8^+^IFNγ^+^ T cells, may have contributed to the superior performance of the S-540956 adjuvant. Supporting the potency of adjuvant S-540956, our results with gD2/S-540956 in the current manuscript outperformed our published results using HSV-2 glycoproteins C, D, and E (gC2, gD2, gE2), trivalent subunit proteins administered with CpG and alum, as a therapeutic vaccine in guinea pigs [33]. 

gD2/S-540956 also outperformed gD2/GLA-SE, a TLR4 agonist. ADCC and T cell ELISPOT responses were significantly higher in the gD2/S-540956-immunized mice than the gD2/GLA-SE mice, which may have contributed to the superior performance of the gD2/S-540956 group. Reagents are limited to measuring CD4 and CD8 T cells, as well as IgG subclass responses, in guinea pigs, rendering it difficult to perform a detailed evaluation of T cells and Th1/Th2 responses in guinea pigs. Our results using gD2/GLA-SE differed from the prior report using GLA-SE with gD2, UL-19, and UL-25, which noted a reduction in recurrent genital lesions, compared to our study, which found no reduction [34]. The inclusion of multiple antigens may have contributed to the efficacy of the trivalent GLA-SE vaccine. The guinea pig model is excellent for assessing the efficacy of therapeutic vaccines; however, better assays to effectively measure T cell responses are needed to define immune correlates of protection.

We used two different immunization schedules. We immunized the animals three times in study 1 and twice in study 2. The vaccine efficacy of gD2/S-540956 was better in study 1 than 2; however, the PBS group had fewer days of HSV-2 DNA shedding in study 1 (16/544 days, 2.9%) than 2 (24/561 days, 4.3%), suggesting that biologic variation between studies may partially explain the difference. In study 1, we detected no increase in serum gD2 IgG titers or neutralizing antibody titers when comparing the first with the third immunization, while in study 2, we observed a small increase when comparing the first immunization with the second. These results suggest that one immunization may be sufficient; however, to further evaluate whether one, two, or three immunizations is preferred, future experiments will need to randomize animals into equal groups based on genital lesions and/or vaginal HSV-2 DNA shedding prior to the second or third immunization. Longer intervals between immunizations may also prove to be beneficial in future guinea pig studies or human trials.

The impressive success using an adjuvanted subunit antigen vaccine to prevent shingles caused by another herpesvirus, varicella-zoster virus, allows for optimism that similar results may be achieved in the treatment of genital herpes [41,42]. Adjuvant S-540956 is the first in its class to be examined with an HSV-2 immunogen as a type of immunotherapy for recurrent genital herpes infections in a guinea pig model. Only a few vaccine candidates for immunotherapy of genital herpes were evaluated in the guinea pig genital infection model prior to human trials [12,34,43,44,45,46,47,48]. None of these candidate vaccines was successful in human trials, and none outperformed gD2/S-540956 in reducing days with genital lesions and vaginal shedding of HSV-2 DNA in the guinea pig model. The immunogens for these studies included gD2 subunit protein with alum; gD2 and gB2 subunit proteins with MF59; gD2, VP11/12, and VP13/14 DNA administered with Vaxfectin; gD2 and ICP4 subunit proteins with MM2; and HSV529 replication defective virus with gD2, UL19, UL25 subunit protein, and GLA-SE [12,34,43,44,45,46,47,48]. A recent mathematical modeling study described the potential benefit of a therapeutic vaccine for genital herpes in an HSV-2/HIV high-prevalence setting. The model predicted that a therapeutic vaccine that is 50% effective and has 40% uptake could reduce the incidence of HSV-2 and HIV by 19% and 17%, respectively, after 40 years if administered to symptomatic individuals with genital herpes [49]. While these numbers are encouraging, we are targeting an efficacy level higher than 50%, and, hopefully, an uptake better than 40%. We consider S-540956 to be an excellent candidate for achieving our goals, particularly if future studies administer S-540956 with multiple HSV-2 antigens, including one or more potent T cell immunogens, and, perhaps, antiviral therapy.

## Figures and Tables

**Table 1:** Vaccine efficacy after 1st immunization, comparing gD2/S-540956 to PBS.

Outcome	Days with Genital Lesions		Days with HSV-2 DNA Shedding		Days with Lesions and/or DNA Shedding	
	PBS	gD2/S ^#^	PBS	gD2/S	PBS	gD2/S
Study 1 3 immunizations	54/720 (7.5%)	21/720 (2.9%)	16/544 (2.9%)	3/544 (0.6%)	70/1264 (5.5%)	24/1264 (1.9%)
Study 1 Vaccine efficacy	61.1%		81.2%		65.7%	
Study 2 2 immunizations	60/829 (7.2%)	30/870 (3.5%)	24/561 (4.3%)	18/590 (3.1%)	84/1390 (6.0%)	48/1460 (3.3%)
Study 2 Vaccine efficacy	52.4%		28.7%		45.7%	
Combined Studies 1 and 2	114/1549 (7.4%)	51/1590 (3.2%)	40/1105 (3.6%)	21/1134 (1.9%)	154/2654 (5.8%)	72/2724 (2.6%)
Combined 1 and 2 Vaccine efficacy	56.4%		48.8%		54.4%	

^#^ gD2/S represents gD2/S-540956. Vaccine efficacy was calculated as: [1 − (outcome in treatment group/outcome in PBS group)] × 100%.

**Table 2:** Animals with total protection against recurrent genital lesions and/or recurrent vaginal shedding of HSV-2 DNA after the 1st immunization, comparing gD2/S-540956 to PBS.

Outcome	Animals with no Recurrent Genital Lesions		Animals with no Recurrent HSV-2 DNA Shedding		Animals with no Genital Lesions and no Shedding	
	PBS	gD2/S ^#^	PBS	gD2/S	PBS	gD2/S
Study 1 3 immunizations	1/16 (6%)	8/16 (50%)	6/16 (38%)	14/16 (88%)	0/16 (0%)	7/16 (44%)
Study 2 2 immunizations	5/19 (26%)	10/20 (50%)	7/19 (37%)	10/20 (50%)	1/19 (5%)	6/20 (30%)
Combined Studies 1 and 2	6/35 (17%)	18/36 (50%)	13/35 (37%)	24/36 (67%)	1/35 (3%)	13/36 (36%)
*p* values combined 1 and 2	0.0054		0.0178		0.0006	

^#^ gD2/S represents gD2/S-540956. *p* values were calculated by the two-tailed Fisher’s exact test.

**Table 3:** Vaccine efficacy after 1st immunization, comparing gD2/S to gD2/ODN and gD2/GLA ^#^.

Outcome	Study 1			Study 2		
	gD2/S	gD2/ODN	Vaccine Efficacy gD2/S vs. gD2/ODN	gD2/S	gD2/GLA	Vaccine Efficacy gD2/S vs. gD2/GLA
Genital lesions	21/720 (2.9%)	33/720 (4.6%)	36.2%	30/870 (3.4%)	89/829 (10.7%)	67.9%
HSV-2 DNA shedding	3/544 (0.6%)	8/544 (1.5%)	62.6%	18/590 (3.1%)	25/561 (4.5%)	31.6%
Combined lesions and shedding	24/1264 (1.9%)	41/1264 (3.2%)	41.4%	48/1460 (3.3%)	114/1390 (8.2%)	59.9%

^#^ gD2/S represents gD2/S-540956; gD2/ODN represents gD2/ODN2006; gD2/GLA represents gD2/GLA-SE. Vaccine efficacy was calculated as: [1 − (outcome in gD2/S group/outcome in gD2/ODN or gD2/GLA group)] × 100%.

## Data Availability

All data reported in the manuscript will be made available upon request from the corresponding authors.

## Supplementary Materials

The following supporting information can be downloaded at: https://www.mdpi.com/article/10.3390/v15051148/s1, Figure S1: Gating strategy to confirm CD8+IFNγ+ responses (% CD8+IFNγ+CTV^−^ cells) analyzed by flow cytometry; Table S1: Vaccine efficacy after 2nd immunization comparing gD2/S-540956 to PBS.
