# Supplementary material for: Novel Adjuvant S-540956 Targets Lymph Nodes and Reduces Genital Recurrences and Vaginal Shedding of HSV-2 DNA When Administered with HSV-2 Glycoprotein D as a Therapeutic Vaccine in Guinea Pigs
Source: Viruses. 2023 May 10;15(5):1148. doi: 10.3390/v15051148 (PMC10220834; doi:10.3390/v15051148)
Supplement: Supplementary file 1 [file viruses-15-01148-s001.zip › viruses-2351315-supplementary.pdf]

Supplementary Materials

Supplementary FigureS1.

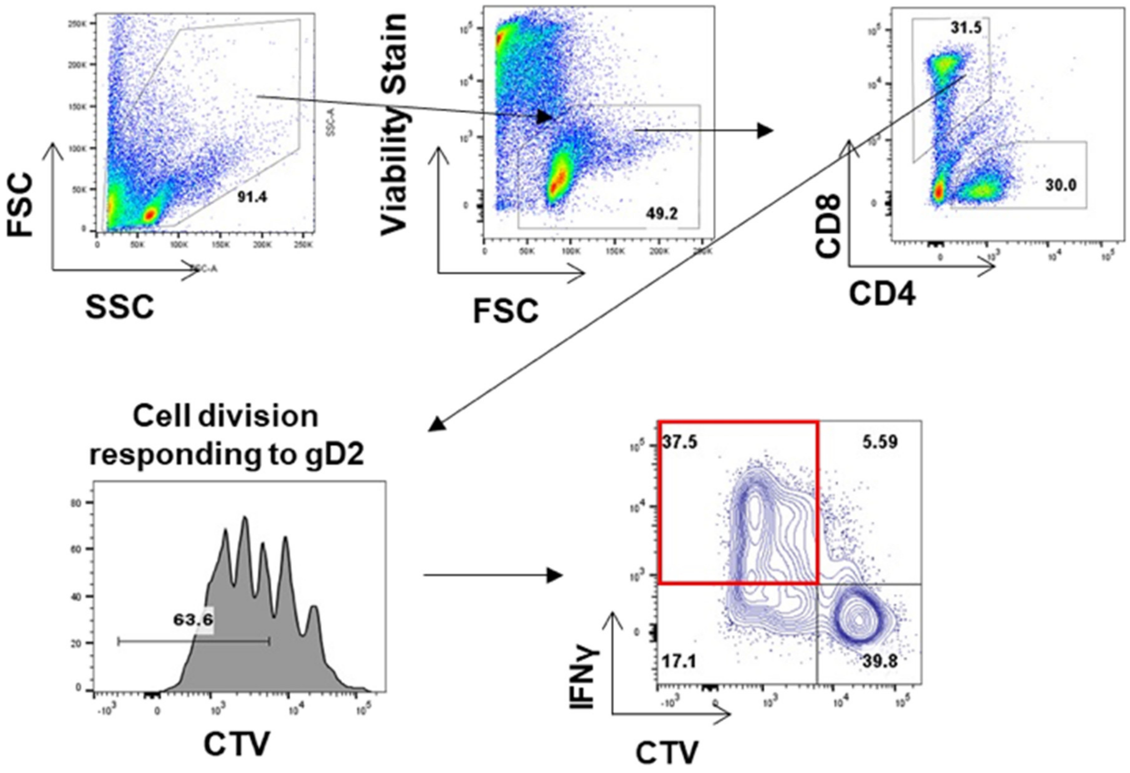

Legend Figure S1. Gating strategy to confirm $CD8^{+}IFN\gamma^{+}$ responses (% $CD8^{+}IFN\gamma^{+}CTV^{-}$ cells) analyzed by flow cytometry.

Supplementary Table S1.

Table S1. Vaccine efficacy after 2nd immunization comparing gD2/S-540956 to PBS

**Outcome**	Days with genital lesions		Days with HSV-2 DNA shedding		Combined days with lesions and/or DNA shedding	
	PBS	gD2/S[#]	PBS	gD2/S	PBS	gD2/S
**Study 1 3 immunizations**	31/560 (5.5%)	11/560 (2.0%)	10/432 (2.3%)	3/432 (0.7%)	41/992 (4.1%)	14/992 (1.4%)
**Study 1 Vaccine efficacy**	64.5%		69.9%		65.8%	
**Study 2 2 immunizations**	41/648 (6.3%)	19/680 (2.8%)	18/466 (3.9%)	15/490 (3.1%)	59/1114 (5.3%)	34/1170 (2.9%)
**Study 2 Vaccine efficacy**	55.9%		20.7%		45.2%	
**Combined Studies 1 & 2**	72/1208 (6.0%)	30/1240 (2.4%)	28/898 (3.1%)	18/922 (2.0%)	100/2106 (4.8%)	48/2162 (2.2%)
**Combined 1 & 2 Vaccine efficacy**	59.4%		37.5%		53.3%	

[#]gD2/S represents gD2/S-540956. Vaccine efficacy was calculated as:
[1 – (outcome in treatment group / outcome in PBS group)] x 100%.
